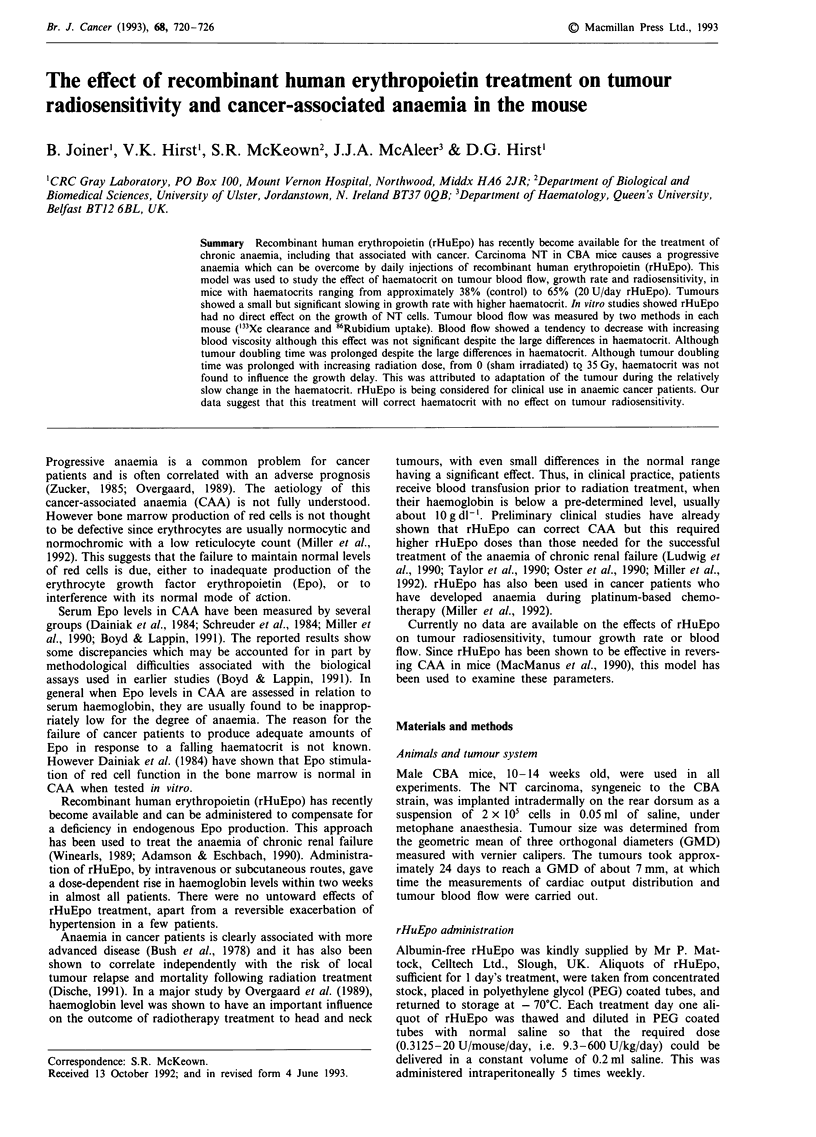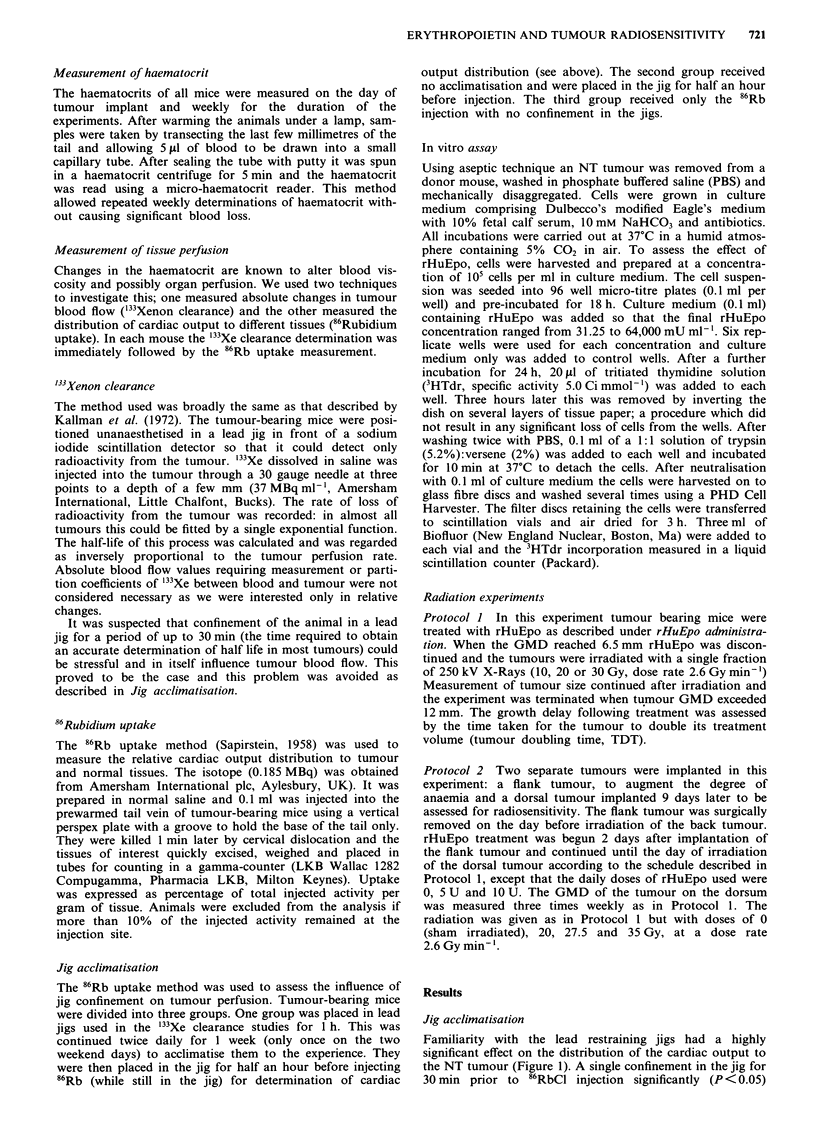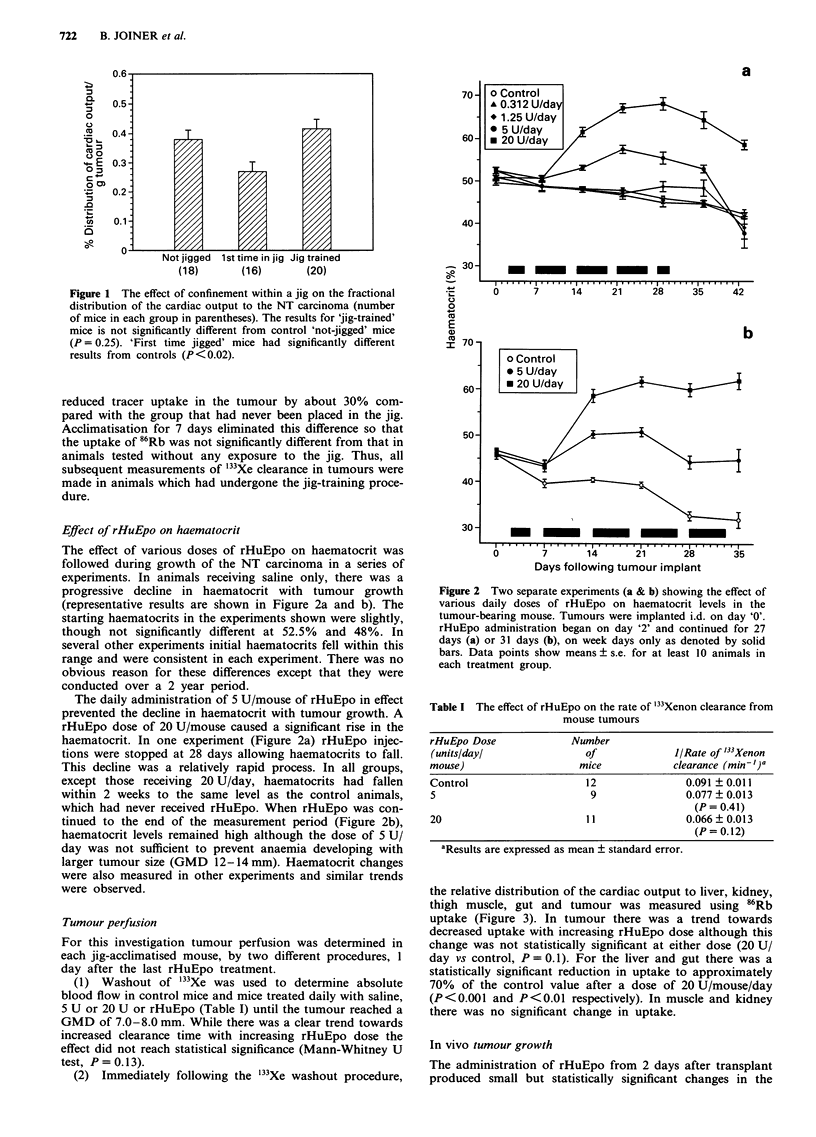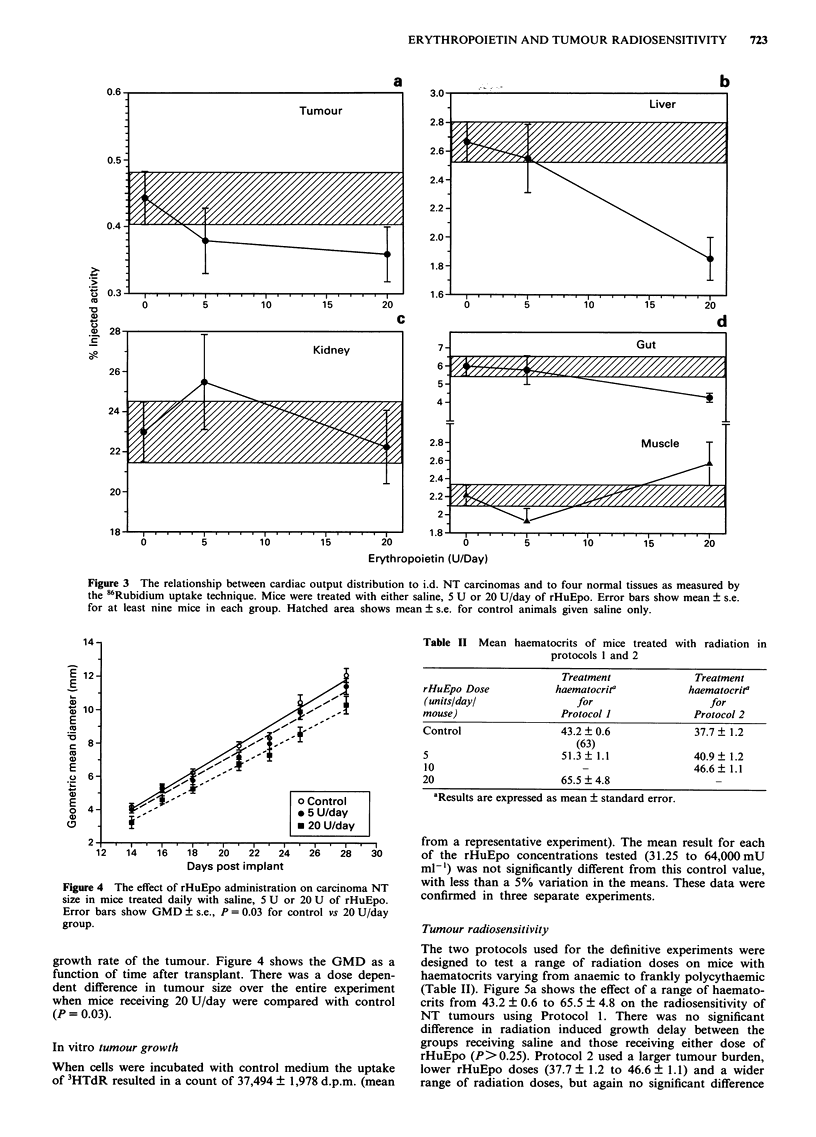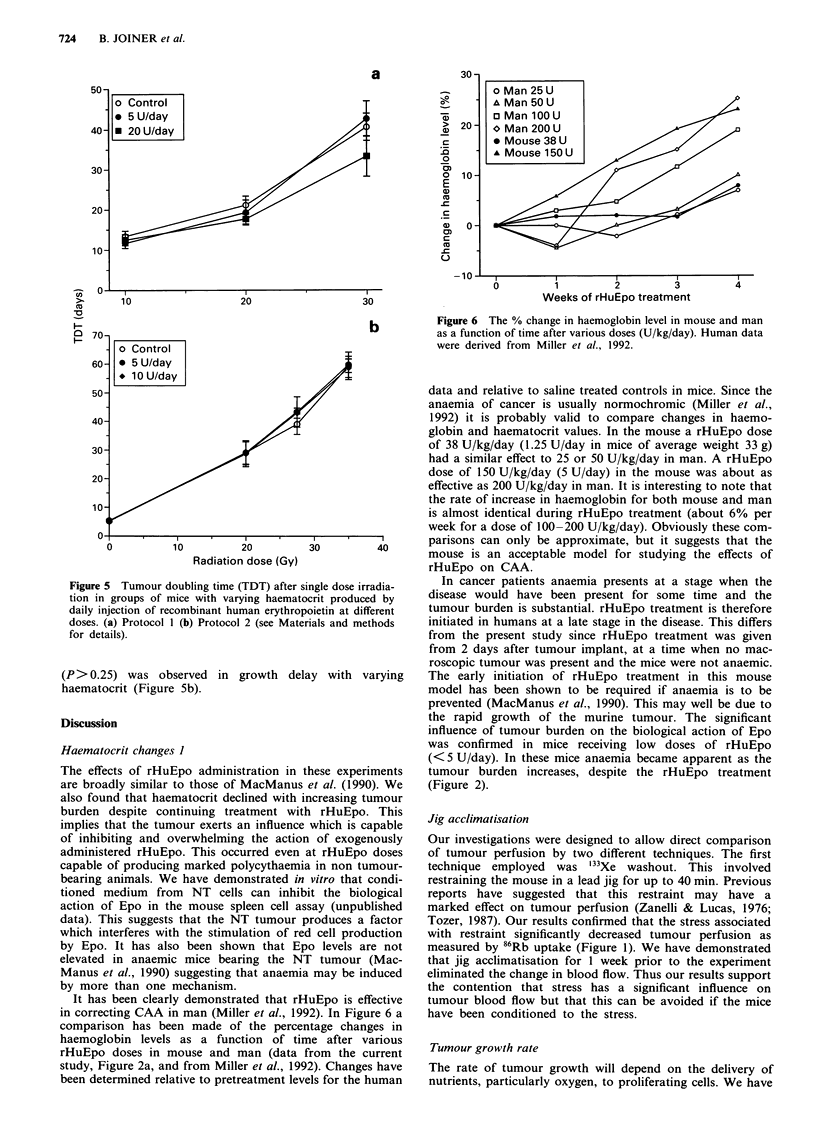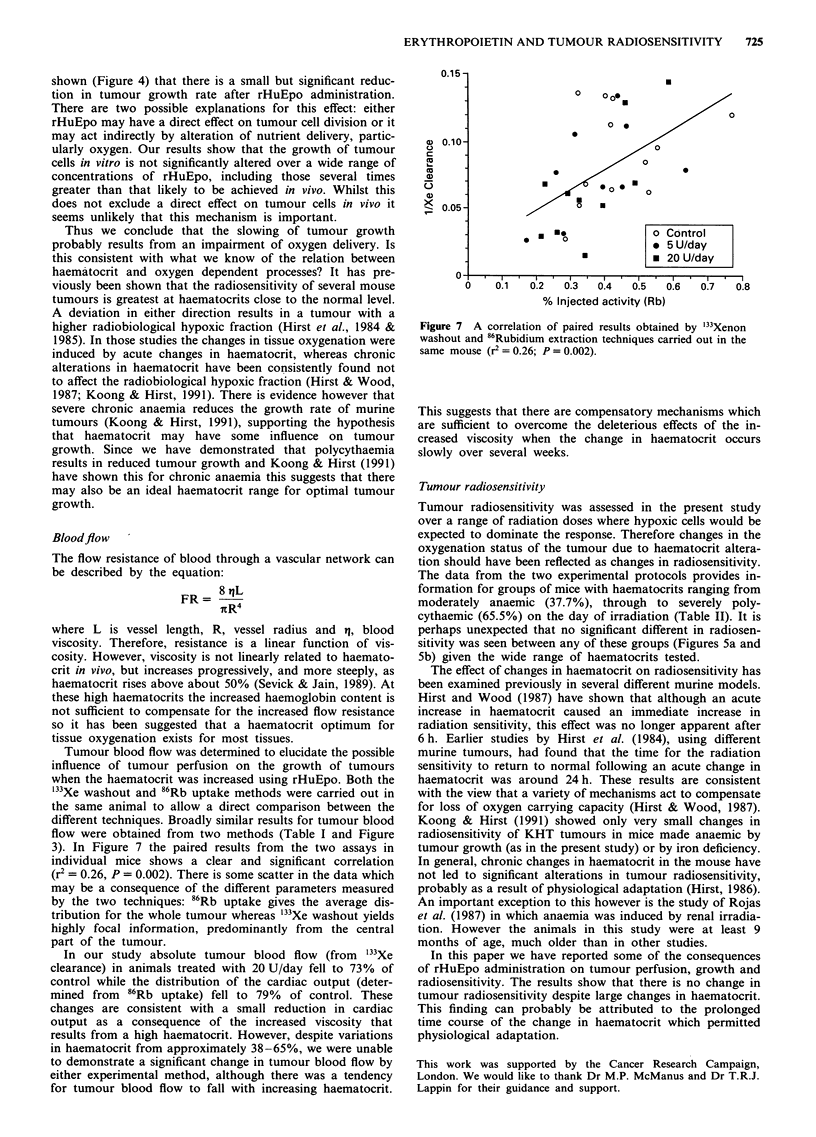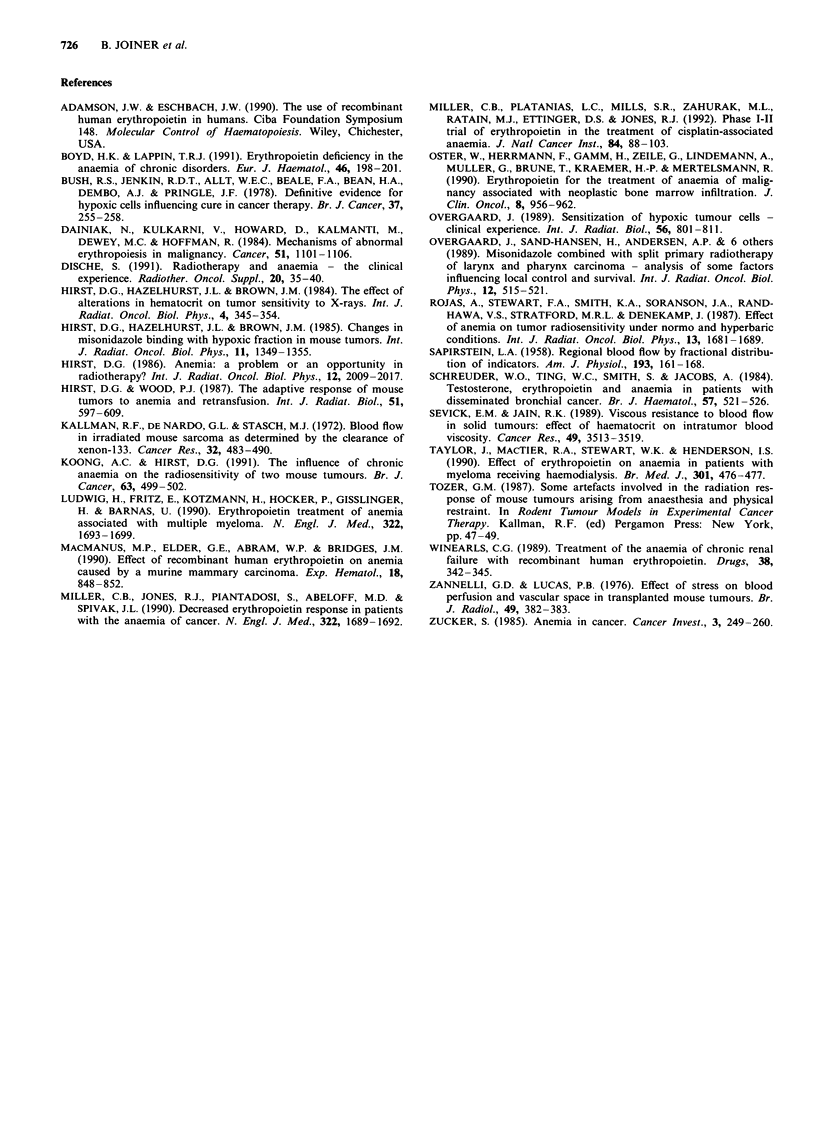# The effect of recombinant human erythropoietin treatment on tumour radiosensitivity and cancer-associated anaemia in the mouse.

**DOI:** 10.1038/bjc.1993.417

**Published:** 1993-10

**Authors:** B. Joiner, V. K. Hirst, S. R. McKeown, J. J. McAleer, D. G. Hirst

**Affiliations:** CRC Gray Laboratory, Mount Vernon Hospital, Northwood, Middx, UK.

## Abstract

Recombinant human erythropoietin (rHuEpo) has recently become available for the treatment of chronic anaemia, including that associated with cancer. Carcinoma NT in CBA mice causes a progressive anaemia which can be overcome by daily injections of recombinant human erythropoietin (rHuEpo). This model was used to study the effect of haematocrit on tumour blood flow, growth rate and radiosensitivity, in mice with haematocrits ranging from approximately 38% (control) to 65% (20 U/day rHuEpo). Tumours showed a small but significant slowing in growth rate with higher haematocrit. In vitro studies showed rHuEpo had no direct effect on the growth of NT cells. Tumour blood flow was measured by two methods in each mouse (133Xe clearance and 86Rubidium uptake). Blood flow showed a tendency to decrease with increasing blood viscosity although this effect was not significant despite the large differences in haematocrit. Although tumour doubling time was prolonged despite the large differences in haematocrit. Although tumour doubling time was prolonged with increasing radiation dose, from 0 (sham irradiated) to 35 Gy, haematocrit was not found to influence the growth delay. This was attributed to adaptation of the tumour during the relatively slow change in the haematocrit. rHuEpo is being considered for clinical use in anaemic cancer patients. Our data suggest that this treatment will correct haematocrit with no effect on tumour radiosensitivity.


Br. J. Cancer (1993), 68, 720-726 © Macmillan Press Ltd., 1993

### The effect of recombinant human erythropoietin treatment on tumour radiosensitivity and cancer-associated anaemia in the mouse

B. Joiner[1], V.K. Hirst[1], S.R. McKeown[2], J.J.A. McAleer[3] & D.G. Hirst[1]

[1]*CRC Gray Laboratory, PO Box 100, Mount Vernon Hospital, Northwood, Middx HA6 2JR;* [2]*Department of Biological and Biomedical Sciences, University of Ulster, Jordanstown, N. Ireland BT37 0QB;* [3]*Department of Haematology, Queen's University, Belfast BT12 6BL, UK.*

**Summary** Recombinant human erythropoietin (rHuEpo) has recently become available for the treatment of chronic anaemia, including that associated with cancer. Carcinoma NT in CBA mice causes a progressive anaemia which can be overcome by daily injections of recombinant human erythropoietin (rHuEpo). This model was used to study the effect of haematocrit on tumour blood flow, growth rate and radiosensitivity, in mice with haematocrits ranging from approximately 38% (control) to 65% (20 U/day rHuEpo). Tumours showed a small but significant slowing in growth rate with higher haematocrit. *In vitro* studies showed rHuEpo had no direct effect on the growth of NT cells. Tumour blood flow was measured by two methods in each mouse ($^{133}$Xe clearance and $^{86}$Rubidium uptake). Blood flow showed a tendency to decrease with increasing blood viscosity although this effect was not significant despite the large differences in haematocrit. Although tumour doubling time was prolonged despite the large differences in haematocrit. Although tumour doubling time was prolonged with increasing radiation dose, from 0 (sham irradiated) to 35 Gy, haematocrit was not found to influence the growth delay. This was attributed to adaptation of the tumour during the relatively slow change in the haematocrit. rHuEpo is being considered for clinical use in anaemic cancer patients. Our data suggest that this treatment will correct haematocrit with no effect on tumour radiosensitivity.

Progressive anaemia is a common problem for cancer patients and is often correlated with an adverse prognosis (Zucker, 1985; Overgaard, 1989). The aetiology of this cancer-associated anaemia (CAA) is not fully understood. However bone marrow production of red cells is not thought to be defective since erythrocytes are usually normocytic and normochromic with a low reticulocyte count (Miller *et al.*, 1992). This suggests that the failure to maintain normal levels of red cells is due, either to inadequate production of the erythrocyte growth factor erythropoietin (Epo), or to interference with its normal mode of action.

Serum Epo levels in CAA have been measured by several groups (Dainiak *et al.*, 1984; Schreuder *et al.*, 1984; Miller *et al.*, 1990; Boyd & Lappin, 1991). The reported results show some discrepancies which may be accounted for in part by methodological difficulties associated with the biological assays used in earlier studies (Boyd & Lappin, 1991). In general when Epo levels in CAA are assessed in relation to serum haemoglobin, they are usually found to be inappropriately low for the degree of anaemia. The reason for the failure of cancer patients to produce adequate amounts of Epo in response to a falling haematocrit is not known. However Dainiak *et al.* (1984) have shown that Epo stimulation of red cell function in the bone marrow is normal in CAA when tested *in vitro*.

Recombinant human erythropoietin (rHuEpo) has recently become available and can be administered to compensate for a deficiency in endogenous Epo production. This approach has been used to treat the anaemia of chronic renal failure (Winearls, 1989; Adamson & Eschbach, 1990). Administration of rHuEpo, by intravenous or subcutaneous routes, gave a dose-dependent rise in haemoglobin levels within two weeks in almost all patients. There were no untoward effects of rHuEpo treatment, apart from a reversible exacerbation of hypertension in a few patients.

Anaemia in cancer patients is clearly associated with more advanced disease (Bush *et al.*, 1978) and it has also been shown to correlate independently with the risk of local tumour relapse and mortality following radiation treatment (Dische, 1991). In a major study by Overgaard *et al.* (1989), haemoglobin level was shown to have an important influence on the outcome of radiotherapy treatment to head and neck tumours, with even small differences in the normal range having a significant effect. Thus, in clinical practice, patients receive blood transfusion prior to radiation treatment, when their haemoglobin is below a pre-determined level, usually about $10\ g\ dl^{-1}$. Preliminary clinical studies have already shown that rHuEpo can correct CAA but this required higher rHuEpo doses than those needed for the successful treatment of the anaemia of chronic renal failure (Ludwig *et al.*, 1990; Taylor *et al.*, 1990; Oster *et al.*, 1990; Miller *et al.*, 1992). rHuEpo has also been used in cancer patients who have developed anaemia during platinum-based chemotherapy (Miller *et al.*, 1992).

Currently no data are available on the effects of rHuEpo on tumour radiosensitivity, tumour growth rate or blood flow. Since rHuEpo has been shown to be effective in reversing CAA in mice (MacManus *et al.*, 1990), this model has been used to examine these parameters.

### Materials and methods

### *Animals and tumour system*

Male CBA mice, 10-14 weeks old, were used in all experiments. The NT carcinoma, syngeneic to the CBA strain, was implanted intradermally on the rear dorsum as a suspension of $2 \times 10^5$ cells in 0.05 ml of saline, under metophane anaesthesia. Tumour size was determined from the geometric mean of three orthogonal diameters (GMD) measured with vernier calipers. The tumours took approximately 24 days to reach a GMD of about 7 mm, at which time the measurements of cardiac output distribution and tumour blood flow were carried out.

### *rHuEpo administration*

Albumin-free rHuEpo was kindly supplied by Mr P. Mattock, Celltech Ltd., Slough, UK. Aliquots of rHuEpo, sufficient for 1 day's treatment, were taken from concentrated stock, placed in polyethylene glycol (PEG) coated tubes, and returned to storage at − 70°C. Each treatment day one aliquot of rHuEpo was thawed and diluted in PEG coated tubes with normal saline so that the required dose (0.3125-20 U/mouse/day, i.e. 9.3-600 U/kg/day) could be delivered in a constant volume of 0.2 ml saline. This was administered intraperitoneally 5 times weekly.

Correspondence: S.R. McKeown.
Received 13 October 1992; and in revised form 4 June 1993.

*Measurement of haematocrit*

The haematocrits of all mice were measured on the day of tumour implant and weekly for the duration of the experiments. After warming the animals under a lamp, samples were taken by transecting the last few millimetres of the tail and allowing 5 µl of blood to be drawn into a small capillary tube. After sealing the tube with putty it was spun in a haematocrit centrifuge for 5 min and the haematocrit was read using a micro-haematocrit reader. This method allowed repeated weekly determinations of haematocrit without causing significant blood loss.

*Measurement of tissue perfusion*

Changes in the haematocrit are known to alter blood viscosity and possibly organ perfusion. We used two techniques to investigate this; one measured absolute changes in tumour blood flow ($^{133}$Xenon clearance) and the other measured the distribution of cardiac output to different tissues ($^{86}$Rubidium uptake). In each mouse the $^{133}$Xe clearance determination was immediately followed by the $^{86}$Rb uptake measurement.

*$^{133}$Xenon clearance*

The method used was broadly the same as that described by Kallman *et al.* (1972). The tumour-bearing mice were positioned unanaesthetised in a lead jig in front of a sodium iodide scintillation detector so that it could detect only radioactivity from the tumour. $^{133}$Xe dissolved in saline was injected into the tumour through a 30 gauge needle at three points to a depth of a few mm (37 MBq ml$^{-1}$, Amersham International, Little Chalfont, Bucks). The rate of loss of radioactivity from the tumour was recorded: in almost all tumours this could be fitted by a single exponential function. The half-life of this process was calculated and was regarded as inversely proportional to the tumour perfusion rate. Absolute blood flow values requiring measurement or partition coefficients of $^{133}$Xe between blood and tumour were not considered necessary as we were interested only in relative changes.

It was suspected that confinement of the animal in a lead jig for a period of up to 30 min (the time required to obtain an accurate determination of half life in most tumours) could be stressful and in itself influence tumour blood flow. This proved to be the case and this problem was avoided as described in *Jig acclimatisation.*

*$^{86}$Rubidium uptake*

The $^{86}$Rb uptake method (Sapirstein, 1958) was used to measure the relative cardiac output distribution to tumour and normal tissues. The isotope (0.185 MBq) was obtained from Amersham International plc, Aylesbury, UK). It was prepared in normal saline and 0.1 ml was injected into the prewarmed tail vein of tumour-bearing mice using a vertical perspex plate with a groove to hold the base of the tail only. They were killed 1 min later by cervical dislocation and the tissues of interest quickly excised, weighed and placed in tubes for counting in a gamma-counter (LKB Wallac 1282 Compugamma, Pharmacia LKB, Milton Keynes). Uptake was expressed as percentage of total injected activity per gram of tissue. Animals were excluded from the analysis if more than 10% of the injected activity remained at the injection site.

*Jig acclimatisation*

The $^{86}$Rb uptake method was used to assess the influence of jig confinement on tumour perfusion. Tumour-bearing mice were divided into three groups. One group was placed in lead jigs used in the $^{133}$Xe clearance studies for 1 h. This was continued twice daily for 1 week (only once on the two weekend days) to acclimatise them to the experience. They were then placed in the jig for half an hour before injecting $^{86}$Rb (while still in the jig) for determination of cardiac output distribution (see above). The second group received no acclimatisation and were placed in the jig for half an hour before injection. The third group received only the $^{86}$Rb injection with no confinement in the jigs.

In vitro *assay*

Using aseptic technique an NT tumour was removed from a donor mouse, washed in phosphate buffered saline (PBS) and mechanically disaggregated. Cells were grown in culture medium comprising Dulbecco's modified Eagle's medium with 10% fetal calf serum, 10 mM $NaHCO_3$ and antibiotics. All incubations were carried out at 37°C in a humid atmosphere containing 5% $CO_2$ in air. To assess the effect of rHuEpo, cells were harvested and prepared at a concentration of $10^5$ cells per ml in culture medium. The cell suspension was seeded into 96 well micro-titre plates (0.1 ml per well) and pre-incubated for 18 h. Culture medium (0.1 ml) containing rHuEpo was added so that the final rHuEpo concentration ranged from 31.25 to 64,000 mU ml$^{-1}$. Six replicate wells were used for each concentration and culture medium only was added to control wells. After a further incubation for 24 h, 20 µl of tritiated thymidine solution ($^3$HTdr, specific activity 5.0 Ci mmol$^{-1}$) was added to each well. Three hours later this was removed by inverting the dish on several layers of tissue paper; a procedure which did not result in any significant loss of cells from the wells. After washing twice with PBS, 0.1 ml of a 1:1 solution of trypsin (5.2%):versene (2%) was added to each well and incubated for 10 min at 37°C to detach the cells. After neutralisation with 0.1 ml of culture medium the cells were harvested on to glass fibre discs and washed several times using a PHD Cell Harvester. The filter discs retaining the cells were transferred to scintillation vials and air dried for 3 h. Three ml of Biofluor (New England Nuclear, Boston, Ma) were added to each vial and the $^3$HTdr incorporation measured in a liquid scintillation counter (Packard).

*Radiation experiments*

*Protocol 1* In this experiment tumour bearing mice were treated with rHuEpo as described under *rHuEpo administration.* When the GMD reached 6.5 mm rHuEpo was discontinued and the tumours were irradiated with a single fraction of 250 kV X-Rays (10, 20 or 30 Gy, dose rate 2.6 Gy min$^{-1}$) Measurement of tumour size continued after irradiation and the experiment was terminated when tumour GMD exceeded 12 mm. The growth delay following treatment was assessed by the time taken for the tumour to double its treatment volume (tumour doubling time, TDT).

*Protocol 2* Two separate tumours were implanted in this experiment: a flank tumour, to augment the degree of anaemia and a dorsal tumour implanted 9 days later to be assessed for radiosensitivity. The flank tumour was surgically removed on the day before irradiation of the back tumour. rHuEpo treatment was begun 2 days after implantation of the flank tumour and continued until the day of irradiation of the dorsal tumour according to the schedule described in Protocol 1, except that the daily doses of rHuEpo used were 0, 5 U and 10 U. The GMD of the tumour on the dorsum was measured three times weekly as in Protocol 1. The radiation was given as in Protocol 1 but with doses of 0 (sham irradiated), 20, 27.5 and 35 Gy, at a dose rate 2.6 Gy min$^{-1}$.

### Results

*Jig acclimatisation*

Familiarity with the lead restraining jigs had a highly significant effect on the distribution of the cardiac output to the NT tumour (Figure 1). A single confinement in the jig for 30 min prior to $^{86}$RbCl injection significantly ($P<0.05$)

**Figure 1** The effect of confinement within a jig on the fractional distribution of the cardiac output to the NT carcinoma (number of mice in each group in parentheses). The results for 'jig-trained' mice is not significantly different from control 'not-jigged' mice ($P = 0.25$). 'First time jigged' mice had significantly different results from controls ($P < 0.02$).

reduced tracer uptake in the tumour by about 30% compared with the group that had never been placed in the jig. Acclimatisation for 7 days eliminated this difference so that the uptake of $^{86}Rb$ was not significantly different from that in animals tested without any exposure to the jig. Thus, all subsequent measurements of $^{133}Xe$ clearance in tumours were made in animals which had undergone the jig-training procedure.

### *Effect of rHuEpo on haematocrit*

The effect of various doses of rHuEpo on haematocrit was followed during growth of the NT carcinoma in a series of experiments. In animals receiving saline only, there was a progressive decline in haematocrit with tumour growth (representative results are shown in Figure 2a and b). The starting haematocrits in the experiments shown were slightly, though not significantly different at 52.5% and 48%. In several other experiments initial haematocrits fell within this range and were consistent in each experiment. There was no obvious reason for these differences except that they were conducted over a 2 year period.

The daily administration of 5 U/mouse of rHuEpo in effect prevented the decline in haematocrit with tumour growth. A rHuEpo dose of 20 U/mouse caused a significant rise in the haematocrit. In one experiment (Figure 2a) rHuEpo injections were stopped at 28 days allowing haematocrits to fall. This decline was a relatively rapid process. In all groups, except those receiving 20 U/day, haematocrits had fallen within 2 weeks to the same level as the control animals, which had never received rHuEpo. When rHuEpo was continued to the end of the measurement period (Figure 2b), haematocrit levels remained high although the dose of 5 U/day was not sufficient to prevent anaemia developing with larger tumour size (GMD 12–14 mm). Haematocrit changes were also measured in other experiments and similar trends were observed.

### *Tumour perfusion*

For this investigation tumour perfusion was determined in each jig-acclimatised mouse, by two different procedures, 1 day after the last rHuEpo treatment.

(1) Washout of $^{133}Xe$ was used to determine absolute blood flow in control mice and mice treated daily with saline, 5 U or 20 U or rHuEpo (Table I) until the tumour reached a GMD of 7.0–8.0 mm. While there was a clear trend towards increased clearance time with increasing rHuEpo dose the effect did not reach statistical significance (Mann-Whitney U test, $P = 0.13$).

(2) Immediately following the $^{133}Xe$ washout procedure,

**Figure 2** Two separate experiments (**a** & **b**) showing the effect of various daily doses of rHuEpo on haematocrit levels in the tumour-bearing mouse. Tumours were implanted i.d. on day '0'. rHuEpo administration began on day '2' and continued for 27 days (**a**) or 31 days (**b**), on week days only as denoted by solid bars. Data points show means ± s.e. for at least 10 animals in each treatment group.

**Table I** The effect of rHuEpo on the rate of $^{133}$Xenon clearance from mouse tumours

| *rHuEpo Dose (units/day/ mouse)* | *Number of mice* | *1/Rate of $^{133}$Xenon clearance ($min^{-1}$)$^a$* |
|---|---|---|
| Control | 12 | 0.091 ± 0.011 |
| 5 | 9 | 0.077 ± 0.013 ($P = 0.41$) |
| 20 | 11 | 0.066 ± 0.013 ($P = 0.12$) |

$^a$Results are expressed as mean ± standard error.

the relative distribution of the cardiac output to liver, kidney, thigh muscle, gut and tumour was measured using $^{86}Rb$ uptake (Figure 3). In tumour there was a trend towards decreased uptake with increasing rHuEpo dose although this change was not statistically significant at either dose (20 U/day *vs* control, $P = 0.1$). For the liver and gut there was a statistically significant reduction in uptake to approximately 70% of the control value after a dose of 20 U/mouse/day ($P < 0.001$ and $P < 0.01$ respectively). In muscle and kidney there was no significant change in uptake.

### In vivo *tumour growth*

The administration of rHuEpo from 2 days after transplant produced small but statistically significant changes in the

**Figure 3** The relationship between cardiac output distribution to i.d. NT carcinomas and to four normal tissues as measured by the $^{86}$Rubidium uptake technique. Mice were treated with either saline, 5 U or 20 U/day of rHuEpo. Error bars show mean ± s.e. for at least nine mice in each group. Hatched area shows mean ± s.e. for control animals given saline only.

**Figure 4** The effect of rHuEpo administration on carcinoma NT size in mice treated daily with saline, 5 U or 20 U of rHuEpo. Error bars show GMD ± s.e., $P = 0.03$ for control *vs* 20 U/day group.

growth rate of the tumour. Figure 4 shows the GMD as a function of time after transplant. There was a dose dependent difference in tumour size over the entire experiment when mice receiving 20 U/day were compared with control ($P = 0.03$).

### In vitro *tumour growth*

When cells were incubated with control medium the uptake of $^{3}$HTdR resulted in a count of 37,494 ± 1,978 d.p.m. (mean from a representative experiment). The mean result for each of the rHuEpo concentrations tested (31.25 to 64,000 mU ml$^{-1}$) was not significantly different from this control value, with less than a 5% variation in the means. These data were confirmed in three separate experiments.

**Table II** Mean haematocrits of mice treated with radiation in protocols 1 and 2

| *rHuEpo Dose (units/day/mouse)* | *Treatment haematocrit[a] for Protocol 1* | *Treatment haematocrit[a] for Protocol 2* |
|---|---|---|
| Control | 43.2 ± 0.6 (63) | 37.7 ± 1.2 |
| 5 | 51.3 ± 1.1 | 40.9 ± 1.2 |
| 10 | – | 46.6 ± 1.1 |
| 20 | 65.5 ± 4.8 | – |

[a]Results are expressed as mean ± standard error.

### *Tumour radiosensitivity*

The two protocols used for the definitive experiments were designed to test a range of radiation doses on mice with haematocrits varying from anaemic to frankly polycythaemic (Table II). Figure 5a shows the effect of a range of haematocrits from 43.2 ± 0.6 to 65.5 ± 4.8 on the radiosensitivity of NT tumours using Protocol 1. There was no significant difference in radiation induced growth delay between the groups receiving saline and those receiving either dose of rHuEpo ($P > 0.25$). Protocol 2 used a larger tumour burden, lower rHuEpo doses (37.7 ± 1.2 to 46.6 ± 1.1) and a wider range of radiation doses, but again no significant difference

**Figure 5** Tumour doubling time (TDT) after single dose irradiation in groups of mice with varying haematocrit produced by daily injection of recombinant human erythropoietin at different doses. (**a**) Protocol 1 (**b**) Protocol 2 (see Materials and methods for details).

($P > 0.25$) was observed in growth delay with varying haematocrit (Figure 5b).

### Discussion

*Haematocrit changes 1*

The effects of rHuEpo administration in these experiments are broadly similar to those of MacManus *et al.* (1990). We also found that haematocrit declined with increasing tumour burden despite continuing treatment with rHuEpo. This implies that the tumour exerts an influence which is capable of inhibiting and overwhelming the action of exogenously administered rHuEpo. This occurred even at rHuEpo doses capable of producing marked polycythaemia in non tumour-bearing animals. We have demonstrated *in vitro* that conditioned medium from NT cells can inhibit the biological action of Epo in the mouse spleen cell assay (unpublished data). This suggests that the NT tumour produces a factor which interferes with the stimulation of red cell production by Epo. It has also been shown that Epo levels are not elevated in anaemic mice bearing the NT tumour (MacManus *et al.*, 1990) suggesting that anaemia may be induced by more than one mechanism.

It has been clearly demonstrated that rHuEpo is effective in correcting CAA in man (Miller *et al.*, 1992). In Figure 6 a comparison has been made of the percentage changes in haemoglobin levels as a function of time after various rHuEpo doses in mouse and man (data from the current study, Figure 2a, and from Miller *et al.*, 1992). Changes have been determined relative to pretreatment levels for the human

**Figure 6** The % change in haemoglobin level in mouse and man as a function of time after various doses (U/kg/day). Human data were derived from Miller *et al.*, 1992.

data and relative to saline treated controls in mice. Since the anaemia of cancer is usually normochromic (Miller *et al.*, 1992) it is probably valid to compare changes in haemoglobin and haematocrit values. In the mouse a rHuEpo dose of 38 U/kg/day (1.25 U/day in mice of average weight 33 g) had a similar effect to 25 or 50 U/kg/day in man. A rHuEpo dose of 150 U/kg/day (5 U/day) in the mouse was about as effective as 200 U/kg/day in man. It is interesting to note that the rate of increase in haemoglobin for both mouse and man is almost identical during rHuEpo treatment (about 6% per week for a dose of 100–200 U/kg/day). Obviously these comparisons can only be approximate, but it suggests that the mouse is an acceptable model for studying the effects of rHuEpo on CAA.

In cancer patients anaemia presents at a stage when the disease would have been present for some time and the tumour burden is substantial. rHuEpo treatment is therefore initiated in humans at a late stage in the disease. This differs from the present study since rHuEpo treatment was given from 2 days after tumour implant, at a time when no macroscopic tumour was present and the mice were not anaemic. The early initiation of rHuEpo treatment in this mouse model has been shown to be required if anaemia is to be prevented (MacManus *et al.*, 1990). This may well be due to the rapid growth of the murine tumour. The significant influence of tumour burden on the biological action of Epo was confirmed in mice receiving low doses of rHuEpo (< 5 U/day). In these mice anaemia became apparent as the tumour burden increases, despite the rHuEpo treatment (Figure 2).

*Jig acclimatisation*

Our investigations were designed to allow direct comparison of tumour perfusion by two different techniques. The first technique employed was $^{133}$Xe washout. This involved restraining the mouse in a lead jig for up to 40 min. Previous reports have suggested that this restraint may have a marked effect on tumour perfusion (Zanelli & Lucas, 1976; Tozer, 1987). Our results confirmed that the stress associated with restraint significantly decreased tumour perfusion as measured by $^{86}$Rb uptake (Figure 1). We have demonstrated that jig acclimatisation for 1 week prior to the experiment eliminated the change in blood flow. Thus our results support the contention that stress has a significant influence on tumour blood flow but that this can be avoided if the mice have been conditioned to the stress.

*Tumour growth rate*

The rate of tumour growth will depend on the delivery of nutrients, particularly oxygen, to proliferating cells. We have shown (Figure 4) that there is a small but significant reduction in tumour growth rate after rHuEpo administration. There are two possible explanations for this effect: either rHuEpo may have a direct effect on tumour cell division or it may act indirectly by alteration of nutrient delivery, particularly oxygen. Our results show that the growth of tumour cells *in vitro* is not significantly altered over a wide range of concentrations of rHuEpo, including those several times greater than that likely to be achieved *in vivo*. Whilst this does not exclude a direct effect on tumour cells *in vivo* it seems unlikely that this mechanism is important.

Thus we conclude that the slowing of tumour growth probably results from an impairment of oxygen delivery. Is this consistent with what we know of the relation between haematocrit and oxygen dependent processes? It has previously been shown that the radiosensitivity of several mouse tumours is greatest at haematocrits close to the normal level. A deviation in either direction results in a tumour with a higher radiobiological hypoxic fraction (Hirst *et al.*, 1984 & 1985). In those studies the changes in tissue oxygenation were induced by acute changes in haematocrit, whereas chronic alterations in haematocrit have been consistently found not to affect the radiobiological hypoxic fraction (Hirst & Wood, 1987; Koong & Hirst, 1991). There is evidence however that severe chronic anaemia reduces the growth rate of murine tumours (Koong & Hirst, 1991), supporting the hypothesis that haematocrit may have some influence on tumour growth. Since we have demonstrated that polycythaemia results in reduced tumour growth and Koong & Hirst (1991) have shown this for chronic anaemia this suggests that there may also be an ideal haematocrit range for optimal tumour growth.

### *Blood flow*

The flow resistance of blood through a vascular network can be described by the equation:

$$FR = \frac{8\,\eta L}{\pi R^4}$$

where L is vessel length, R, vessel radius and $\eta$, blood viscosity. Therefore, resistance is a linear function of viscosity. However, viscosity is not linearly related to haematocrit *in vivo*, but increases progressively, and more steeply, as haematocrit rises above about 50% (Sevick & Jain, 1989). At these high haematocrits the increased haemoglobin content is not sufficient to compensate for the increased flow resistance so it has been suggested that a haematocrit optimum for tissue oxygenation exists for most tissues.

Tumour blood flow was determined to elucidate the possible influence of tumour perfusion on the growth of tumours when the haematocrit was increased using rHuEpo. Both the $^{133}$Xe washout and $^{86}$Rb uptake methods were carried out in the same animal to allow a direct comparison between the different techniques. Broadly similar results for tumour blood flow were obtained from two methods (Table I and Figure 3). In Figure 7 the paired results from the two assays in individual mice shows a clear and significant correlation ($r^2 = 0.26$, $P = 0.002$). There is some scatter in the data which may be a consequence of the different parameters measured by the two techniques: $^{86}$Rb uptake gives the average distribution for the whole tumour whereas $^{133}$Xe washout yields highly focal information, predominantly from the central part of the tumour.

In our study absolute tumour blood flow (from $^{133}$Xe clearance) in animals treated with 20 U/day fell to 73% of control while the distribution of the cardiac output (determined from $^{86}$Rb uptake) fell to 79% of control. These changes are consistent with a small reduction in cardiac output as a consequence of the increased viscosity that results from a high haematocrit. However, despite variations in haematocrit from approximately 38–65%, we were unable to demonstrate a significant change in tumour blood flow by either experimental method, although there was a tendency for tumour blood flow to fall with increasing haematocrit.

**Figure 7** A correlation of paired results obtained by $^{133}$Xenon washout and $^{86}$Rubidium extraction techniques carried out in the same mouse ($r^2 = 0.26$; $P = 0.002$).

This suggests that there are compensatory mechanisms which are sufficient to overcome the deleterious effects of the increased viscosity when the change in haematocrit occurs slowly over several weeks.

### *Tumour radiosensitivity*

Tumour radiosensitivity was assessed in the present study over a range of radiation doses where hypoxic cells would be expected to dominate the response. Therefore changes in the oxygenation status of the tumour due to haematocrit alteration should have been reflected as changes in radiosensitivity. The data from the two experimental protocols provides information for groups of mice with haematocrits ranging from moderately anaemic (37.7%), through to severely polycythaemic (65.5%) on the day of irradiation (Table II). It is perhaps unexpected that no significant different in radiosensitivity was seen between any of these groups (Figures 5a and 5b) given the wide range of haematocrits tested.

The effect of changes in haematocrit on radiosensitivity has been examined previously in several different murine models. Hirst and Wood (1987) have shown that although an acute increase in haematocrit caused an immediate increase in radiation sensitivity, this effect was no longer apparent after 6 h. Earlier studies by Hirst *et al.* (1984), using different murine tumours, had found that the time for the radiation sensitivity to return to normal following an acute change in haematocrit was around 24 h. These results are consistent with the view that a variety of mechanisms act to compensate for loss of oxygen carrying capacity (Hirst & Wood, 1987). Koong & Hirst (1991) showed only very small changes in radiosensitivity of KHT tumours in mice made anaemic by tumour growth (as in the present study) or by iron deficiency. In general, chronic changes in haematocrit in the mouse have not led to significant alterations in tumour radiosensitivity, probably as a result of physiological adaptation (Hirst, 1986). An important exception to this however is the study of Rojas *et al.* (1987) in which anaemia was induced by renal irradiation. However the animals in this study were at least 9 months of age, much older than in other studies.

In this paper we have reported some of the consequences of rHuEpo administration on tumour perfusion, growth and radiosensitivity. The results show that there is no change in tumour radiosensitivity despite large changes in haematocrit. This finding can probably be attributed to the prolonged time course of the change in haematocrit which permitted physiological adaptation.

This work was supported by the Cancer Research Campaign, London. We would like to thank Dr M.P. McManus and Dr T.R.J. Lappin for their guidance and support.

### References

ADAMSON, J.W. & ESCHBACH, J.W. (1990). The use of recombinant human erythropoietin in humans. Ciba Foundation Symposium 148. *Molecular Control of Haematopoiesis*. Wiley, Chichester, USA.

BOYD, H.K. & LAPPIN, T.R.J. (1991). Erythropoietin deficiency in the anaemia of chronic disorders. *Eur. J. Haematol.*, **46**, 198–201.

BUSH, R.S., JENKIN, R.D.T., ALLT, W.E.C., BEALE, F.A., BEAN, H.A., DEMBO, A.J. & PRINGLE, J.F. (1978). Definitive evidence for hypoxic cells influencing cure in cancer therapy. *Br. J. Cancer*, **37**, 255–258.

DAINIAK, N., KULKARNI, V., HOWARD, D., KALMANTI, M., DEWEY, M.C. & HOFFMAN, R. (1984). Mechanisms of abnormal erythropoiesis in malignancy. *Cancer*, **51**, 1101–1106.

DISCHE, S. (1991). Radiotherapy and anaemia – the clinical experience. *Radiother. Oncol. Suppl.*, **20**, 35–40.

HIRST, D.G., HAZELHURST, J.L. & BROWN, J.M. (1984). The effect of alterations in hematocrit on tumor sensitivity to X-rays. *Int. J. Radiat. Oncol. Biol. Phys.*, **4**, 345–354.

HIRST, D.G., HAZELHURST, J.L. & BROWN, J.M. (1985). Changes in misonidazole binding with hypoxic fraction in mouse tumors. *Int. J. Radiat. Oncol. Biol. Phys.*, **11**, 1349–1355.

HIRST, D.G. (1986). Anemia: a problem or an opportunity in radiotherapy? *Int. J. Radiat. Oncol. Biol. Phys.*, **12**, 2009–2017.

HIRST, D.G. & WOOD, P.J. (1987). The adaptive response of mouse tumors to anemia and retransfusion. *Int. J. Radiat. Biol.*, **51**, 597–609.

KALLMAN, R.F., DE NARDO, G.L. & STASCH, M.J. (1972). Blood flow in irradiated mouse sarcoma as determined by the clearance of xenon-133. *Cancer Res.*, **32**, 483–490.

KOONG, A.C. & HIRST, D.G. (1991). The influence of chronic anaemia on the radiosensitivity of two mouse tumours. *Br. J. Cancer*, **63**, 499–502.

LUDWIG, H., FRITZ, E., KOTZMANN, H., HOCKER, P., GISSLINGER, H. & BARNAS, U. (1990). Erythropoietin treatment of anemia associated with multiple myeloma. *N. Engl. J. Med.*, **322**, 1693–1699.

MACMANUS, M.P., ELDER, G.E., ABRAM, W.P. & BRIDGES, J.M. (1990). Effect of recombinant human erythropoietin on anemia caused by a murine mammary carcinoma. *Exp. Hematol.*, **18**, 848–852.

MILLER, C.B., JONES, R.J., PIANTADOSI, S., ABELOFF, M.D. & SPIVAK, J.L. (1990). Decreased erythropoietin response in patients with the anaemia of cancer. *N. Engl. J. Med.*, **322**, 1689–1692.

MILLER, C.B., PLATANIAS, L.C., MILLS, S.R., ZAHURAK, M.L., RATAIN, M.J., ETTINGER, D.S. & JONES, R.J. (1992). Phase I-II trial of erythropoietin in the treatment of cisplatin-associated anaemia. *J. Natl Cancer Inst.*, **84**, 88–103.

OSTER, W., HERRMANN, F., GAMM, H., ZEILE, G., LINDEMANN, A., MULLER, G., BRUNE, T., KRAEMER, H.-P. & MERTELSMANN, R. (1990). Erythropoietin for the treatment of anaemia of malignancy associated with neoplastic bone marrow infiltration. *J. Clin. Oncol.*, **8**, 956–962.

OVERGAARD, J. (1989). Sensitization of hypoxic tumour cells – clinical experience. *Int. J. Radiat. Biol.*, **56**, 801–811.

OVERGAARD, J., SAND-HANSEN, H., ANDERSEN, A.P. & 6 others (1989). Misonidazole combined with split primary radiotherapy of larynx and pharynx carcinoma – analysis of some factors influencing local control and survival. *Int. J. Radiat. Oncol. Biol. Phys.*, **12**, 515–521.

ROJAS, A., STEWART, F.A., SMITH, K.A., SORANSON, J.A., RANDHAWA, V.S., STRATFORD, M.R.L. & DENEKAMP, J. (1987). Effect of anemia on tumor radiosensitivity under normo and hyperbaric conditions. *Int. J. Radiat. Oncol. Biol. Phys.*, **13**, 1681–1689.

SAPIRSTEIN, L.A. (1958). Regional blood flow by fractional distribution of indicators. *Am. J. Physiol.*, **193**, 161–168.

SCHREUDER, W.O., TING, W.C., SMITH, S. & JACOBS, A. (1984). Testosterone, erythropoietin and anaemia in patients with disseminated bronchial cancer. *Br. J. Haematol.*, **57**, 521–526.

SEVICK, E.M. & JAIN, R.K. (1989). Viscous resistance to blood flow in solid tumours: effect of haematocrit on intratumor blood viscosity. *Cancer Res.*, **49**, 3513–3519.

TAYLOR, J., MACTIER, R.A., STEWART, W.K. & HENDERSON, I.S. (1990). Effect of erythropoietin on anaemia in patients with myeloma receiving haemodialysis. *Br. Med. J.*, **301**, 476–477.

TOZER, G.M. (1987). Some artefacts involved in the radiation response of mouse tumours arising from anaesthesia and physical restraint. In *Rodent Tumour Models in Experimental Cancer Therapy*. Kallman, R.F. (ed) Pergamon Press: New York, pp. 47–49.

WINEARLS, C.G. (1989). Treatment of the anaemia of chronic renal failure with recombinant human erythropoietin. *Drugs*, **38**, 342–345.

ZANNELLI, G.D. & LUCAS, P.B. (1976). Effect of stress on blood perfusion and vascular space in transplanted mouse tumours. *Br. J. Radiol.*, **49**, 382–383.

ZUCKER, S. (1985). Anemia in cancer. *Cancer Invest.*, **3**, 249–260.